# Viral and Atypical Bacterial Etiology of Acute Respiratory Infections in Children under 5 Years Old Living in a Rural Tropical Area of Madagascar

**DOI:** 10.1371/journal.pone.0043666

**Published:** 2012-08-17

**Authors:** Jonathan Hoffmann, Henintsoa Rabezanahary, Martin Randriamarotia, Arsène Ratsimbasoa, Josette Najjar, Guy Vernet, Bénédicte Contamin, Gláucia Paranhos-Baccalà

**Affiliations:** 1 Centre d'Infectiologie Charles Mérieux (CICM), Faculty of Medicine, Antananarivo, Madagascar; 2 Fondation Mérieux, Antananarivo, Madagascar; 3 Fondation Médicale d'Ampasimanjeva (FMA), Ampasimanjeva, Madagascar; 4 Ministère de la Santé Publique, Antanananarivo, Madagascar; 5 Emerging Pathogens Laboratory, Fondation Mérieux, SFR BioSciences Gerland – Lyon Sud (UMS3444/US8), Lyon, France; Louisiana State University Health Sciences Center, United States of America

## Abstract

**Background:**

In Madagascar, very little is known about the etiology and prevalence of acute respiratory infections (ARIs) in a rural tropical area. Recent data are needed to determine the viral and atypical bacterial etiologies in children with defined clinical manifestations of ARIs.

**Methods:**

During one year, we conducted a prospective study on ARIs in children between 2 to 59 months in the community hospital of Ampasimanjeva, located in the south-east of Madagascar. Respiratory samples were analyzed by multiplex real-time RT-PCR, including 18 viruses and 2 atypical bacteria. The various episodes of ARI were grouped into four clinical manifestations with well-documented diagnosis: “Community Acquired Pneumonia”(CAP, group I), “Other acute lower respiratory infections (Other ALRIs, group II)”, “Upper respiratory tract infections with cough (URTIs with cough, group III)”and “Upper respiratory tract infections without cough (URTIs without cough, group IV)”.

**Results:**

295 children were included in the study between February 2010 and February 2011. Viruses and/or atypical bacteria respiratory pathogens were detected in 74.6% of samples, the rate of co-infection was 27.3%. Human rhinovirus (HRV; 20.5%), metapneumovirus (HMPV A/B, 13.8%), coronaviruses (HCoV, 12.5%), parainfluenza virus (HPIV, 11.8%) and respiratory syncytial virus A and B (RSV A/B, 11.8%) were the most detected. HRV was predominantly single detected (23.8%) in all the clinical groups while HMPV A/B (23.9%) was mainly related to CAP (group I), HPIV (17.3%) to the “Other ALRIs” (group II), RSV A/B (19.5%) predominated in the group “URTIs with cough” (group III) and Adenovirus (HAdV, 17.8%) was mainly detected in the “without cough” (group IV).

**Interpretation:**

This study describes for the first time the etiology of respiratory infections in febrile children under 5 years in a malaria rural area of Madagascar and highlights the role of respiratory viruses in a well clinically defined population of ARIs.

## Introduction

Acute respiratory infections (ARIs) are a major public health problem causing approximately 1.9 million child deaths in 2000 [1]. About 20% of child mortality (<5 years) is due to pneumonia, bronchitis or bronchiolitis and 90% of them are attributed to pneumonia [2]. Community acquired pneumonia (CAP) is a major cause of morbidity and hospitalization in developed countries and a major cause of mortality among children living in developing countries [3] where socio-economic issues such as malnutrition are aggravating factors. To date, it is difficult to reliably predict the pathogen based on clinical signs and symptoms [4]. Respiratory pathogens etiologies could help to better understand acute febrile illnesses in malaria endemic area and orient adapted therapies. Viruses were considered as causative agents of acute lower respiratory infections (ALRIs) and have been investigated in several studies [5]. Since 2001, several new respiratory viruses have been described such as metapneumovirus (HMPV) [6], human coronavirus (HCoV), NL63 [7] and HKU1 [8] and human bocavirus (HBoV) [9]. Various respiratory viruses that caused epidemics and pandemic, such as swine lineage influenza ALRIs A (H1N1) virus infection, in 2009, have heightened the need to develop sensitive and rapid diagnostic test. The development of molecular methods such as multiplex Real-Time PCR (RT-PCR) greatly facilitates the etiological study of respiratory infections but it does not, especially in developing countries, assist the clinician in the care of patient. Madagascar is a country with low HIV prevalence (estimated at 0.2% in 2010 [10]) and malaria is endemic with stable transmission during all year on the East Coast. Recent studies on surveillance of fever among child result in malaria over-diagnosis with consequent under diagnosis of other fever-causing disorders such as pneumonia [11]. Little is known about the pathogens responsible for ARIs especially in rural areas. A recent study shows that respiratory viruses play an important role in children under 5 years old consulting in public and private clinics in Antananarivo with Influenza-Like Illnesses (ILIs) symptoms [12]. In Ampasimanjeva, a small village located in a rural area endemic for malaria, a recent study showed that 68% of acute fever illnesses among children are not explained by malaria (Ratsimbasoa, personal communication). The objective of this study is to determine the prevalence and seasonal distribution of a large panel of respiratory pathogens including viruses and atypical bacteria among a well clinically defined cohort of acute febrile children between 2 to 59 months of age presenting clinical ARIs symptoms in Ampasimanjeva.

**Table 1:** Pathogens prevalence among outpatient children aged from 2 to 59 months living in Ampasimanjeva district between February 2010 and February 2011.

Pathogen identified	Single detection	Co-detection	Total
	n (%)	n (%)	n (%)
HRV	38 (23.8)	23 (16.8)	61 (20.5)
HMPV A/B	31 (19.4)	10 (7.3)	41 (13.8)
HCoV	15 (9.4)	22 (16.1)	37 (12.5)
HCoV-NL63	2 (1.3)	9 (6.6)	11 (3.7)
HCoV-OC43	11 (6.9)	10 (7.3)	21 (7.1)
HCoV-229E	2 (1.3)	0	2 (0.7)
HCoV-HKU1	0	3 (2.2)	3 (1.0)
RSV A/B	24 (15.0)	11 (8.0)	35 (11.8)
HPIV	13 (8.1)	22 (16.1)	35 (11.8)
HPIV−2	1 (0.6)	5 (3.6)	6 (2.0)
HPIV−3	11 (6.9)	11 (8.0)	22 (7.4)
HPIV−4	1 (0.6)	6 (4.4)	7 (2.4)
FLUV	20 (12.5)	6 (4.4)	26 (8.8)
FLUAV	9 (5.6)	4 (2.9)	13 (4.4)
FLUAV (H1N1/pdm09)	4 (2.5)	1 (0.7)	5 (1.7)
FLUBV	7 (4.4)	1 (0.7)	8 (2.7)
HAdV	9 (5.6)	10 (7.3)	19 (6.4)
HBoV	1 (0.6)	17 (12.4)	18 (6.1)
EV	4 (2.5)	13 (9.5)	17 (5.7)
*Mpneu*	3 (1.9)	2 (1.5)	5 (1.7)
*Cpneu*	2 (1.3)	0	2 (0.7)
PV	0	1 (0.7)	1 (0.3)
**Total pathogens identified**	**160** (100.0)	**137** (100.0)	**297** (100.0)
**Sample tested**			
Positive samples	160 (72.7)	60 (27.3)	220 (74.6)
Negative samples	-	-	75 (25.4)
**Total samples**	-	-	**295** (100.0)

**Table 2:** Co-occurrence of respiratory pathogens in nasopharyngeal samples from children with ARIs.

Primary viral agent	Associated respiratory pathogens	n
HBoV	HRV	6
	HCoV	5
	EV	4
	HPIV	3
	HAdV	3
	HMPV	2
	RSV A/B	1
	*Mpneu*	1
	**Total**	**25**
**HPIV**	HCoV	9
	HRV	5
	RSV A/B	3
	HAdV	3
	HBoV	3
	EV	1
	**Total**	**24**
**HCoV**	HPIV	9
	HRV	6
	HBoV	5
	RSV A/B	4
	EV	3
	FLUV	2
	**Total**	**29**
**HRV**	EV	7
	HBoV	6
	HCoV	6
	HPIV	5
	RSV A/B	2
	HMPV	2
	HAdV	2
	FLUV	2
	**Total**	**32**

Total represents the number of co-detected pathogens from each primary viral agent.

## Methods

### Ethics statement

This study was approved by the National Ethics Committee of the Malagasy Ministry of Health (CE/MINSAN n° 019). A briefing note explaining the purpose of the project and the informed consent form was given to each of the parents involved in the study who signed the consent forms to provide written informed consent.

### Study subjects and specimens

In October 2009, community health workers and physicians carried out a census of all children under 5 years old living in the rural community of Ampasimanjeva. Between February 2010 and February 2011, each child presenting an axillary temperature >37.5°C or a history of fever within the previous 24 hours was followed-up by physicians. First, each febrile child was evaluated with a rapid diagnostic test for malaria. Those positive for malaria (n = 21) were treated with Artemisinin-based combination therapy (ACT) and were not enrolled in the study. Those with a negative test for malaria (n = 916) and presenting symptoms of acute respiratory infections (n = 295) defined by the presence of Ear-Nose-Throat (ENT) signs or auscultatory signs were included in the study. Among the 295 children enrolled, 24.1% (71/295) had taken an antipyretic treatment and 6.8% (20/295) had declared taking antibiotics before consultation. After completing a full clinical record, a nasopharyngeal swab sample was performed on each child enrolled. The swab was placed in a universal transport medium (UTM, Copan) and stored at 4°C until sent to the Centre d'Infectiologie Charles Mérieux at Antananarivo, Madagascar for analysis. Pending the molecular diagnostic, biological samples were stored at −80°C.

**Figure 1:**
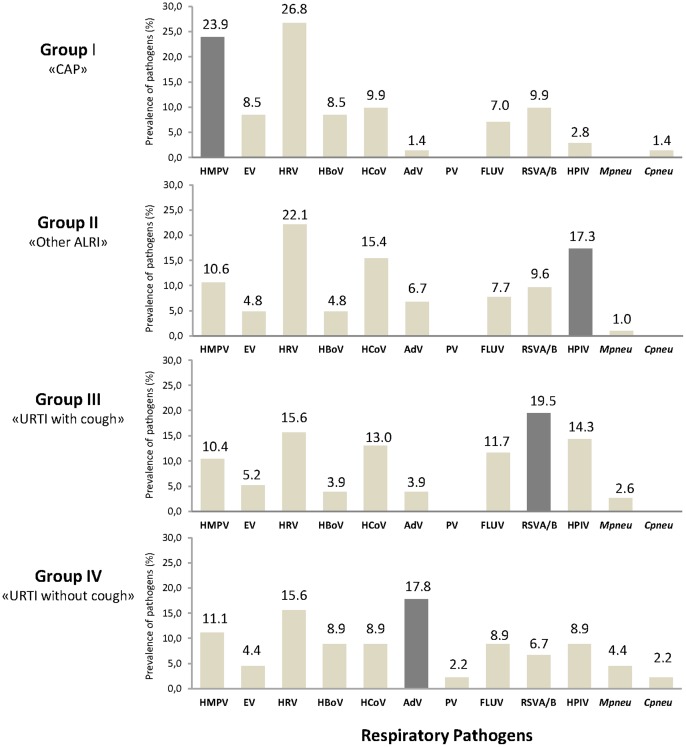
ARIs viral and atypical bacterial etiologies related to clinical manifestation groups. All 295 acute respiratory infection episodes were classified in 4 clinical manifestation groups (Group I, n = 83; Group II, n = 95, Group III, n = 66; group IV, n = 51 cases). The respiratory pathogens number upon the bar represents the percentage of the total pathogens identified in each clinical group. The dark grey bars correspond to the significant association (*P* = 0.05) between the pathogen and the clinical group.

### Clinical manifestations of ARIs

Clinical records provided by physicians were classified in four clinical ARIs episodes: non-severe “Community Acquired Pneumonia” (**Group I**: “CAP”) were defined according to WHO criteria, i.e in children with fever (axillary temperature>37.5°C) and tachypnea (pulsation rate >50 per minute in a child aged 2 −11 months and >40 per minute in child aged ≥12 months). Chest indrawing were considered as a severity factor of pneumonia (severe “Community Acquired”). A child with fever and at least one auscultator's signs including crackles, rhonchi and/or wheeze was considered in the **Group II** (“Other”) including bronchiolitis, bronchitis and episodic viral wheeze/asthma (EVW). Upper respiratory tract infections (URTIs) were diagnosed based on symptoms such as cough, runny nose or ear and/or nasopharyngitis and related to **Group III** (“URTIs with cough”) and to **Group IV** (“URTIs without cough”).

**Table 3:** Relationship between child temperature and ARI viral and bacterial etiologies.

Child temperature	[37.5°C −39°C]	[>39°C]
	n (%)	n (%)
HRV	54 (88.5)	7 (11.5)
HMPV A/B	32 (78.0)	9 (22.0)
HCoV	30 (81.1)	7 (18.9)
RSV A/B	27 (77.1)	8 (22.9)
HPIV	28 (80.0)	7 (20.0)
FLUV	17 (65.4)	9 (34.6)
HAdV	17 (89.5)	2 (10.5)
HBoV	14 (77.8)	4 (22.2)
EV	14 (82.4)	3 (17.6)
*Mpneu*	1 (20)	4 (80)
*Cpneu*	2 (100)	0
PV	1 (0.4)	0
Total pathogens identified	**237**	**60**
Total PCR positive cases	175 (74.5)	45 (75.0)
Total PCR negative cases	60 (25.5)	15 (25.0)
**Total cases**	**235**	**60**

Percentage in brackets represents the number of evaluated samples in each child temperature group.

### Setting and study design

This is a descriptive and prospective study performed on children under 5 years of age consulting at Ampasimanjeva community hospital with clinical symptoms of ARIs between February 2010 and February 2011. Ampasimanjeva is located in the south-east of Madagascar, in a tropical malaria endemic region where two seasons exist: hot and rainy between November and April, cold and dry between May and October. The proposed study covers both seasonal periods.

### PCR analyses

Nucleic acids were extracted from biological samples (nasopharyngeal swab) with Qiamp RNA Virus Kit (Qiagen) following the protocol provided by the manufacturer. Multiplex real-time PCR assays were performed with FTD Respiratory 21 PLUS pathogens panel (Fast-Track Diagnosis, Luxembourg). This kit allows the identification of major respiratory pathogens (2 atypical bacteria and 18 viruses) including: Influenza viruses A and B (FLUAV and FLUBV) and Influenza A virus subtype H1N1 2009 [FLUAV (H1N1/pdm09)], human coronaviruses: NL63 (HCoV-NL63), 229E (HCoV-229E), OC43 (HCoV-OC43) and HKU1 (HCoV-HKU1), human parainfluenza (HPIV-1, −2, −3, −4), human metapneumovirus A and B (HMPV A/B), human rhinovirus (HRV), respiratory syncytial virus A and B (RSV A/B), human adenovirus (HAdV), enterovirus (EV), parechovirus (PV), human bocavirus (HBoV), Mycoplasma pneumoniae (Mpneu), and Chlamydia pneumoniae (Cpneu). Internal control was added to each analysis. All PCR assays were performed with the AgPath-IDTM One-Step RT PCR kit (Ambion, cat#AM1005) as recommended by FTD company. For some analyses, the following pathogens were grouped: HCoV-OC43, HCoV-HKU1, HCoV-229E and HCoV-NL63 in human coronaviruses (HCoV group); FLUAV, FLUBV, (FLUAV/H1N1/pdm09) in Influenza virus (FLUV group); HPIV−2, HPIV−3 and HPIV−4 in human Parainfluenza virus (HPIV group).

**Figure 2:**
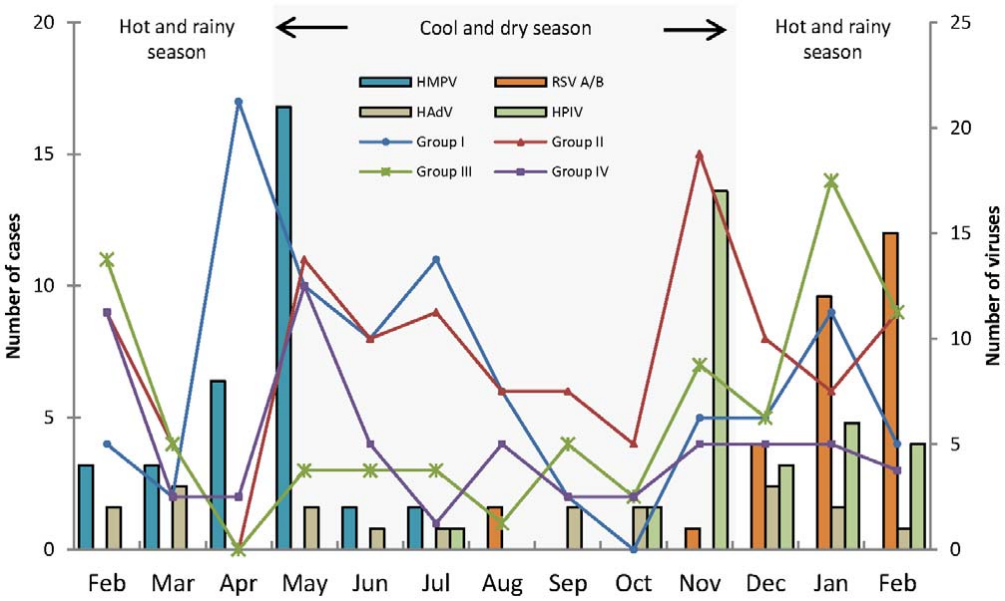
Seasonal distribution of acute respiratory infections in children, by type of the most frequently detected pathogens during one year. The number of cases collected in each defined clinical groups were represented on the left and the number of the viruses found were represented on the right.

### Statistical analysis of data

Associations between clinical manifestations and etiological agents were analyzed with Fisher Exact test, considering a P-value <0.05 as significant value. Statistical analyses were calculated with EpiInfo software (version 3.5.1).

## Results

### Study population

Between February 2010 and February 2011, 937 febrile children aged between 24 to 59 months were recorded and followed-up by physicians, 21 were malaria positive and 961 were malaria negative. Among malaria negative children, 295 were enrolled in the study and 60 presented acute fever (axillary temperature >39°C). A significant remaining part of children with fever and negative tested for malaria (n = 642) was not included in the study. The mean age of the children was 26.4 months (median age of 24 months). Considering clinical records provided by physicians, children were classified into 4 groups: 83 (28%) in group I (“CAP”), 95 (32%) in group II (“other ALRIs”), 66 (22%) in group III (“URTIs with”) and 51 (18%) in group IV (“URTIs without”). The following clinical manifestations were considered: nasopharyngitis (271/295, 92%), cough (197/295, 67%), crackles (87/295, 29%), rhonchi (74/295, 25%), wheeze (22/295, 7%), chest indrawing (16/295, 5%) and stridor (2/295, 1%).

### Viral and atypical bacterial etiologies

Out of 295 biological samples tested, 220 (75%) were positive for at least 1 respiratory pathogen. Among the 220 positive cases, 297 pathogens were identified as single or dual-detections. Table 1 summarized the pathogens prevalence found in the Ampasimanjeva children samples. HRV, HMPV A/B, HCoV, RSV A/B and HPIV were the commonest pathogens identified. In the human coronaviruses and parainfluenza families, HCoV-OC43 and HPIV−3 were the most detected. No HPIV−1 infection was identified. Single detection was commonly observed in 160 specimens and caused mainly by HRV (23.8% of all single detected pathogen), HMPV A/B (19.4%), RSV A/B (15.0%) and FLUV (12.5%) whereas co-detections were found in 60 samples (27.3%). Dual detection was observed in 43 samples and three pathogens were found in 17 samples mostly related to HRV, HCoV, HBoV, and HPIV (Table 2). No co-detection with more than three pathogens was observed. The co-detection between HCoV and HPIV represents the major association observed in this study (n = 9). Age was not a risk factor that can impact in the pathogen identification into the defined clinical groups.

### Respiratory pathogens related to the clinical manifestations of ARIs

In this study, most pathogens were found distributed among the four groups representing the different clinical manifestations (Figure 1). Overall, HRV was detected in each group presenting a highest prevalence in ALRIs (26.8% and 22.1% in group I and II, respectively) compared to the others groups. HMPV A/B was mainly detected among “CAP” patients (17/71, *P* = 0.020) and most often single detected (15/17). HPIV was associated with bronchiolitis, bronchitis and EVW (18/104, *P* = 0.021). RSV A/B was commonly observed among children having Flu-like illnesses (Group III, 15/77, P = 0.022). HAdV infections were frequently associated with “URTI without cough” (8/45, *P*<0.01). Severe community acquired pneumonia represented 17% of the group I (14 out of 83) including 10 positive cases for at least 1 pathogen, such as HMPV A/B (n = 4), HRV (n = 3), HBoV (n = 3), EV (n = 3) and *C*pneu (n = 1).

### Fever consideration

Overall, 220 out 295 cases (74.6%), positive for at least one respiratory pathogen, presented an axillary temperature above 37.5°C during the study inclusion and sample collection. Moreover, among the 220 cases, 60 children (20.3%) presented a temperature above 39°C. These 60 cases were mostly represented in the ALRIs (groups I and II, data not shown) and 75% were positive for at least one etiological agent with a predominance of Influenza and HMP viruses. However, when we compared the two fever sub-groups, the Influenza virus is the pathogen most represented (15%, *P* = 0.055) in the group with acute fever (temperature >39°C). Interestingly, 80% (n = 4/5) of *Mycoplasma pneumoniae* detected were associated with acute fever (*P-*value insignificant). The remaining respiratory pathogens were detected in patient with mild fever (Table 3).

### Seasonal distribution

In the Figure 2, the seasonality of the mainly respiratory pathogens found in each defined clinical group was represented. The HMPV circulated from February to July with a peak in April/May 2010 corresponding to the end of the hot and rainy season. This season is correlated to the highest number of CAP cases (group I). The end of the cool and dry season was correlated to the peak of the other ALRIs cases (group II), where we observed the HPIV and HCoV co-circulation (data not shown). RSV A/B infection shows a significant seasonal variation with a peak in the month of February 2011, during the hot and rainy season correlating with Flu-like illnesses cases (group III) and Influenza virus infection peaks (data not shown). The beginning of the cool and dry season represents the seasonality where we found the largest number of cases in each defined clinical group, except for the group III. Finally, HRV (data not shown) and HAdV circulated throughout the year without any epidemic peak.

## Discussion

This study describes for the first time the etiology of a clinically defined cohort of children with fever and acute respiratory infections living in a malaria endemic rural region of Madagascar. The rural community of Ampasimanjeva consists of seven villages located in south-eastern Madagascar (coordinates 21° 44 ′0 “South/48° 2′ 0” East) dominated by an equatorial climate with high humidity. Recently, 1,549 children under 5 years of age were referenced and among them, 937 presented a fever and were examined at the hospital during the study period. Of these children, 295 presented ARIs clinical signs and a negative test for malaria. To better understand the role of the viral and atypical bacteria agents in nasopharyngeal swabs, we stratified the respiratory clinical symptoms into 4 major groups to obtain defined ARIs cohorts. Seventy five percent (n = 220) of the acute febrile children had a nasopharyngeal sample containing at least one virus or atypical bacterial agent. The low rate of negative specimens shows that the technique used in this study is highly sensitive and the spectrum of pathogens is large, covering most of those implicated in the ARIs. In this study, the frequently encountered pathogens were the HRV, HMPV A/B, HCoV, RSV A/B and HPIV. The most representative pathogens into the defined clinical groups were HMPV, HPIV, HRSV and HAdV for pneumonia, other ALRIs, Flu-like illnesses and URTI, respectively. The human rhinovirus, HMPV A/B and RSV A/B were single detected while HCoV, HPIV and HBoV were most often co-detected. Human rhinovirus was found largely in all clinical manifestations.

HRV has long been considered to be a benign virus causing mild upper respiratory tract infections, but there is evidence that HRV is also involved in ALRIs [13] more specifically in bronchiolitis [14], but its role is not yet well defined in pneumonia [15], [16]. HRV is frequently found as asymptomatic carriage [17], [18], [19], a comprehensive study on genotypes from HRV-positive samples would be interesting. As, it seems that the new and potentially more pathogenic HRV-C which has been recently discovered [20], [21] is correlated with the severity of ARIs [22], [23].

Our molecular results showed a large number of respiratory pathogens associated with the different clinical manifestations including a large part in pneumonia. Viral pneumonia is also increasingly described in the literature but it is nevertheless still underestimated [3], [19]. In children, RSV, HRV and HMPV have become important pathogens most frequently encountered in children with pneumonia in developed countries [24]–[27]. In Ampasimanjeva, although detected in all ARIs clinical manifestations, the human metapneumovirus was better associated with community-acquired pneumonia with a higher prevalence than those described in the literature [24], [28], [29]. HMPV is, however, considered to have an important role in pneumonia increasing child morbidity worldwide [25], [30]. In a study done in Antananarivo (2008/2009) [12], RSV A/B was mainly represented among children with Influenza-Like Illnesses and similarly, our results showed that this virus was primarily associated with group II which we defined here as Influenza-like syndrome.

Atypical bacteria pathogens were also investigated. We detected a low rate of *M. pneumoniae* (1.7%) and *C. pneumoniae* (0.7%) distributed in all four clinical groups. Our results correlate to those described in several studies, which show that atypical bacteria prevalence in acute respiratory infections may vary depending on the age of children [28], [31]–[33].

Our study has several limitations. Firstly, we did not investigate the viral and atypical respiratory pathogens in control population as asymptomatic carriers. This could limit our interpretation to ascribe the etiological agents to the defined clinical groups. However, some of the potential etiological agents described here are rarely identified or less common in asymptomatic patients, except for rhinoviruses [19], [34], [35]. A longitudinal case/control study would be interesting to validate the relevance of these agents in acute respiratory infections despite the difficulty to obtain approval requirements from ethical committee. Our results highlight a significant proportion of viruses among children with ARIs. However, the viral etiology of an infection does not exclude the coexistence of a bacterial infection nor superinfection, often observed, for example, with pneumococci following influenza virus infection [36]. The bacterial etiology could not be evaluated in this study but remains important enough to be investigated further. Using information gathered from clinical records, it appears that 87.5% of children received antibiotics as a result of auscultation. If only viruses are actually mostly the cause of ALRIs in Ampasimanjeva, simple recommendations such as those proposed by WHO and UNICEF [37] could reduce the morbidity and mortality. Secondly, we did not evaluate the proportion of respiratory pathogens among children with malaria (n = 21). In the absence of appropriate diagnostic tools in low income countries, integrated management of childhood illnesses (IMCI) at health facilities is presumptive and symptom-based: fever for malaria, and fever/cough/difficult breathing for pneumonia. These overlapping symptoms, compatible both with malaria and pneumonia, necessitating dual treatment, need to be evaluated in further studies. Unexplained acute febrile illness is the only common factor in children living in endemic malaria region. In this study, we showed that the fever cannot be considered alone to guide clinical diagnosis. Moreover, respiratory pathogens do not seem to be related to fever prognosis except for Influenza viruses. Finally, the interpretation of the fever may be biased by the use of antipyretic treatment, and 24.1% of our patients had evidence of antipyretic use before study enrollment. Finally, this study was conducted over one year, covering both hot/rainy and cold/dry seasons, to get a preliminary description of respiratory pathogens and respiratory pathogens spread during this period. This short time period should be prolonged to deepen our knowledge about these pathogens and perhaps anticipate epidemics in light of improving the health care of children.

In conclusion, the use of molecular assays has allowed us to refine our understanding of the viral etiologies of ARIs among children living in a rural area of Madagascar. Further studies are needed to properly distinguish between infection and colonization. They will lead to comparing findings in different respiratory samples and reference standards. Viruses seem to be commonly involved in pneumonia. While some viruses such as HRV are mainly represented in all clinical presentations, some such as hMPV and RSV are most often associated with pneumonia or influenza-like illnesses. A better understanding of the biodiversity of each pathogen correlated to well-defined clinical ARIs manifestations could be explored.

## References

[1] Williams BG, Gouws E, Boshi- Pinto C, Bryce J, Dye C (2002) Estimates of world-wide distribution of child deaths from acute respiratory infections. Lancet Infectious Disease 2: 25–32. 10.1016/s1473-3099(01)00170-0 11892493

[2] World Health Organization (2012) “Acute respiratory infections in children”. Available: http://www.who.int/vaccine_research/diseases/ari/en/index.html. Accessed 2012 Mar 27.

[3] Mc Cracken GH (2000) Diagnosis and management of pneumonia in children. Pediatr Infect Dis J 19: 924–928. 11001128 10.1097/00006454-200009000-00036

[4] Caliendo AM (2011) Multiplex PCR and Emerging Technologies for the Detection of Respiratory Pathogens. Clinical Infectious Diseases 52 (S4): S326–S330. 10.1093/cid/cir047 PMC7107927 21460291

[5] Pavia AT (2011) Viral infections of the lower respiratory tract: old viruses, new viruses, and the role of diagnosis. Clin Infect Dis. May 52 Suppl 4S284–9. 10.1093/cid/cir043 PMC3106235 21460286

[6] Van den Hoogen BG, de Jong JC, Groen J, Kuiken T, de Groot R, et al (2001) A newly discovered human pneumovirus isolated from young children with respiratory tract disease. Nat Med. Jun 7(6): 719–24. 10.1038/89098 PMC7095854 11385510

[7] Van der Hoek L, Ihorst G, Sure K, Vabret A, Dijkman R, et al (2010) Burden of disease due to human coronavirus NL63 infections and periodicity of infection. J Clin Virol. Jun 48(2): 104–8. 10.1016/j.jcv.2010.02.023 PMC7108429 20347384

[8] Woo PC, Lau SK, Chu CM, Chan KH, Tsoi HW, et al (2005) Characterization and complete genome sequence of a novel coronavirus, coronavirus HKU1, from patients with pneumonia. J Virol. Jan 79(2): 884–95. 10.1128/JVI.79.2.884-895.2005 PMC538593 15613317

[9] Allander T, Tammi MT, Eriksson M, Bjerkner A, Tiveljung-Lindell A, et al (2005) Cloning of a human parvovirus by molecular screening of respiratory tract samples. Proc Natl Acad Sci U S A. Sep 6 102(36): 12891–6. 10.1073/pnas.0504666102 PMC1200281 16118271

[10] ONUAIDS (2010) “UNAIDS Report on the Global AIDS Epidemic”. Available: http://www.unaids.org/globalreport/global_report.htm. Accessed 2012 Mar 27.

[11] Amexo M, Tolhurst R, Barnish G, Bates I (2004) Malaria misdiagnosis: effects on the poor and vulnerable. Lancet. Nov 20–26 364(9448): 1896–8. 10.1016/S0140-6736(04)17446-1 15555670

[12] Razanajatovo NH, Richard V, Hoffmann J, Reynes JM, Razafitrimo GM, et al (2011) Viral etiology of influenza-like illnesses in Antananarivo, Madagascar, July 2008 to June 2009. PLoS One. Mar 3 6(3): e17579. 10.1371/journal.pone.0017579 PMC3048401 21390235

[13] Papadopoulos NG, Bates PJ, Bardin PG, Papi A, Leir SH, et al (2000) Rhinoviruses infect the lower airways. J Infect Dis. Jun 181(6): 1875–84. 10.1086/315513 10837165

[14] Richard N, Komurian-Pradel F, Javouhey E, Perret M, Rajoharison A, et al (2008) The impact of dual viral infection in infants admitted to a pediatric intensive care unit associated with severe bronchiolitis. Pediatr Infect Dis J. Mar 27(3): 213–7. 10.1097/INF.0b013e31815b4935 18277932

[15] Broberg E, Niemelä J, Lahti E, Hyypiä T, Ruuskanen O, et al (2011) Human rhinovirus C--associated severe pneumonia in a neonate.J Clin Virol. May 51(1): 79–82. 10.1016/j.jcv.2011.01.018 PMC7172304 21342784

[16] Tapparel C, L'Huillier AG, Rougemont AL, Beghetti M, Barazzone-Argiroffo C, et al (2009) Pneumonia and pericarditis in a child with HRV-C infection: a case report. J Clin Virol. Jun 45(2): 157–60. 10.1016/j.jcv.2009.03.014 PMC7108322 19427260

[17] Mackay IM (2008) Human rhinoviruses: the cold wars resume. J Clin Virol. Aug 42(4): 297–320. 10.1016/j.jcv.2008.04.002 PMC7108405 18502684

[18] Jartti T, Jartti L, Peltola V, Waris M, Ruuskanen O (2008) Identification of respiratory viruses in asymptomatic subjects: asymptomatic respiratory viral infections. Pediatr Infect Dis J. Dec 27(12): 1103–7. 10.1097/INF.0b013e31817e695d 18978518

[19] Mermond(2012) Lower Respiratory Infections Among Hospitalized Children in New Caledonia: A Pilot Study for the Pneumonia Etiology Research for Child Health Project. Clin Infect Dis. 54 22)S180–S189 http://cid.oxfordjournals.org/content/54/suppl_2/S180.short – aff-1 S, Zurawskihttp://cid.oxfordjournals.org/content/54/suppl_2/S180.short – aff-1 V, D’Ortenzio E, Driscollhttp://cid.oxfordjournals.org/content/54/suppl_2/S180.short – aff-3 AJ, DeLucahttp://cid.oxfordjournals.org/content/54/suppl_2/S180.short – aff-3 AN, et al . 10.1093/cid/cir1070 PMC7107894 22403234

[20] Lamson D, Renwick N, Kapoor V, Liu Z, Palacios G, et al (2006) MassTag polymerase-chain-reaction detection of respiratory pathogens, including a new rhinovirus genotype, that caused influenza-like illness in New York State during 2004–2005. J Infect Dis. 2006 Nov 15 194(10): 1398–402. 10.1086/508551 PMC7110122 17054069

[21] Kaiser L, Aubert JD, Pache JC, Deffernez C, Rochat T, et al. (2006) Chronic rhinoviral infection in lung transplant recipients. Am J Respir Crit Care Med. Dec 15;174(12):1392–9. Epub 2006 Sep 28. 10.1164/rccm.200604-489OC 17008640

[22] Renwick N, Schweiger B, Kapoor V, Liu Z, Villari J, et al (2007) A recently identified rhinovirus genotype is associated with severe respiratory-tract infection in children in Germany. J Infect Dis. Dec 15 196(12): 1754–60. 10.1086/524312 PMC7109967 18190255

[23] Brownlee JW, Turner RB (2008) New developments in the epidemiology and clinical spectrum of rhinovirus infections. Curr Opin Pediatr 20: 67–71. 18197042 10.1097/MOP.0b013e3282f41cb6

[24] Cevey-Macherel M, Galetto-Lacour A, Gervaix A, Siegrist C-A, Bille J, et al (2009) Etiology of community-acquired pneumonia in hospitalized children based on WHO clinical guidelines. Eur J Pediatr 168: 1429–1436. 19238436 10.1007/s00431-009-0943-y PMC7087130

[25] Ruuskanen O, Lahti E, Jennings LC, Murdoch DR (2011) Viral pneumonia. Lancet 377: 1264–75. 21435708 10.1016/S0140-6736(10)61459-6 PMC7138033

[26] Honkinen M, Lahti E, Osterback R, Ruuskanen O, Waris M (2011) Viruses and bacteria in sputum samples of children with community-acquired pneumonia. Clin Microbiol Infect. Jun 14. doi: 10.1111/j.1469–0691.2011.03603.x. 10.1111/j.1469-0691.2011.03603.x PMC7128628 21851481

[27] Tsolia MN, Psarras S, Bossios A, Audi H, Paldanius M, et al (2004) Etiology of Community-Acquired Pneumonia in Hospitalized School-Age Children: Evidence for High Prevalence of Viral Infections. Clinical Infectious Diseases 39: 681–686. 15356783 10.1086/422996 PMC7107828

[28] Bezerra PG, Britto MC, Correia JB, Duarte Mdo C, Fonceca AM, et al (2011) Viral and atypical bacterial detection in acute respiratory infection in children under five years. PLoS One. Apr 18 6(4): e18928. 10.1371/journal.pone.0018928 PMC3078930 21533115

[29] Do AH, van Doorn HR, Nghiem MN, Bryant JE, Hoang TH, et al (2011) Viral etiologies of acute respiratory infections among hospitalized Vietnamese children in Ho Chi Minh City, 2004–2008. PLoS One. Mar 24 6(3): e18176. 10.1371/journal.pone.0018176 PMC3063798 21455313

[30] Nascimento-Carvalho CM, Cardoso MR, Ruuskanen O, Lappalainen M (2011) Sole infection by human metapneumovirus among children with radiographically diagnosed community-acquired pneumonia in a tropical region. Influenza Other Respi Viruses. Jul 5(4): 285–7 doi: 10.1111/j.1750–2659.2011.00206.x. 10.1111/j.1750-2659.2011.00206.x PMC4634542 21651739

[31] García Ramos E, Pizarro Suárez E, Sapiáin LA, Lugo de la Fuente G (1991) Epidemiologic and etiologic study of acute respiratory infections in children under 5 years of age. Rev Latinoam Microbiol. Apr-Sep 33(2–3): 109–19. 1670472

[32] Saikku P, Ruutu P, Leinonen M, Kleemola M, Paladin F, et al (1993) Mycoplasma pneumoniae and Chlamydia trachomatis in acute lower respiratory infections in Filipino children. Am J Trop Med Hyg. Jul 49(1): 88–92. 10.4269/ajtmh.1993.49.88 8352396

[33] Blic J, Delacourt C (2009) Pneumologie pédiatrique. Flammarion Médecine-Sciences. p69.

[34] Palma SC, Martínez TMA, Salinas SM, Rojas GP (2005) Asymptomatic pharyngeal carriage of Mycoplasma pneumoniae in Chilean children. Rev Chilena Infectol. Sep 22(3): 247–50. 10.4067/s0716-10182005000300005 16077892

[35] Kumar S, Wang L, Fan J, Kraft A, Bose ME, et al (2008) Detection of 11 common viral and bacterial pathogens causing community-acquired pneumonia or sepsis in asymptomatic patients by using a multiplex reverse transcription-PCR assay with manual (enzyme hybridization) or automated (electronic microarray) detection. J Clin Microbiol. Sep 46(9): 3063–72. 10.1128/JCM.00625-08 PMC2546717 18650351

[36] O'Brien KL, Walters MI, Sellman J, Quinlisk P, Regnery H, et al (2000) Severe pneumococcal pneumonia in previously healthy children: the role of preceding influenza infection. Clin Infect Dis. May 30(5): 784–9. 10.1086/313772 10816149

[37] World Health Organisation, UNICEF (2008) “Global Action Plan for Prevention and Control of Pneumonia (GAPP)”. Available: http://www.unicef.org/media/files/GAPP3_web.pdf. Accessed 2012 Mar 27.

